# Baseline predictors of early progression during EGFR-TKI therapy in patients with EGFR-mutant non-small cell lung cancer: A retrospective cohort study

**DOI:** 10.1097/MD.0000000000049948

**Published:** 2026-07-31

**Authors:** Xiaoyan Guo, Likai Liu, Hongyan Yu, Shifeng Zhao, Chunyan Wang, Xiansheng Wang

**Affiliations:** a Respiratory and Critical Care Medicine Department II, Affiliated Hospital of Hebei University of Engineering, Handan City, Hebei Province, China; b Emergency Department I, Affiliated Hospital of Hebei University of Engineering, Handan City, Hebei Province, China.

**Keywords:** early progression, EGFR-mutant non-small cell lung cancer, EGFR tyrosine kinase inhibitor, metastatic pattern, multivariable logistic regression, RECIST, tumor burden

## Abstract

This single-center retrospective cohort study investigated baseline clinical, molecular, and treatment-related factors associated with early progression among patients with epidermal growth factor receptor (EGFR)-mutant non-small cell lung cancer receiving EGFR tyrosine kinase inhibitors (EGFR-TKIs) between June 2022 and June 2025. Eligible patients treated during the study period were consecutively screened according to predefined eligibility criteria, and all eligible patients were included without case-control matching. Early progression was defined a priori as progressive disease within 6 months after EGFR-TKI initiation according to response evaluation criteria in solid tumors version 1.1; this cutoff was selected as a clinically meaningful landmark for early treatment failure/primary resistance and to distinguish rapid failure from patients achieving at least short-term disease control. A total of 156 patients were included (early progression, n = 52; nonearly progression, n = 104). Compared with nonearly progression, early progression was characterized by a different smoking distribution, lower body mass index, higher prevalence of chronic obstructive pulmonary disease, and greater baseline tumor burden as reflected by a higher number of target lesions, as well as higher rates of liver metastasis, bone metastasis, and pleural effusion. Molecular and treatment profiling showed higher frequencies of TP53 co-mutation and baseline EGFR T790M in early progressors, together with a higher proportion of first-generation EGFR-TKI use and more frequent receipt of EGFR-TKI as second-line or later therapy. In multivariable logistic regression, current smoking, lower body mass index, higher target lesion count, liver metastasis, bone metastasis, pleural effusion, second-line or later EGFR-TKI use, first-generation EGFR-TKI exposure, and mesenchymal–epithelial transition factor amplification were independently associated with early progression, while baseline EGFR T790M showed a borderline association. These findings support risk-adapted monitoring and molecularly informed management strategies in EGFR-mutant non-small cell lung cancer.

## 1. Introduction

Epidermal growth factor receptor (EGFR) mutations define a major actionable subset of non-small cell lung cancer (NSCLC), for which EGFR tyrosine kinase inhibitors (EGFR-TKIs) have become foundational systemic therapy. Contemporary treatment algorithms recommend osimertinib as a preferred option for patients with advanced EGFR exon 19 deletion or exon 21 L858R-mutant NSCLC, reflecting its established clinical activity and central nervous system penetration.^[1,2]^ Despite these advances, clinically meaningful heterogeneity in treatment benefit persists, and a subset of patients experiences early disease progression after initiation of targeted therapy. Real-world studies further indicate that progression patterns after first-line osimertinib are variable and can differ by clinical context, emphasizing the need for refined risk stratification beyond EGFR driver status alone.^[3,4]^

Early progression under EGFR-TKI therapy is clinically consequential because it may reflect intrinsic resistance, high baseline tumor burden, or the presence of co-occurring molecular alterations that diminish EGFR dependency. Prospective and translational investigations have characterized diverse resistance mechanisms to osimertinib, including activation of bypass signaling pathways and molecular heterogeneity between tissue and plasma, underscoring that resistance can be present at baseline or emerge rapidly under treatment selection pressure.^[5,6]^ In parallel, emerging mechanistic data support a role for perturbations in cell-cycle regulation in intrinsic osimertinib resistance, providing a biological rationale for why some patients progress early despite exposure to a third-generation EGFR-TKI.^[7]^ As the therapeutic landscape evolves, including the development of next-generation inhibitors designed to address resistant EGFR variants, the clinical utility of baseline predictors that identify patients at risk of early failure remains high.^[8]^ Beyond tumor genomics, host-related factors and disease burden metrics may influence early outcomes. Nutritional status and body composition abnormalities have been increasingly recognized in NSCLC populations receiving oral targeted agents, with evidence indicating that sarcopenia and related phenotypes are prevalent and clinically relevant during EGFR-TKI therapy.^[9,10]^ These observations support the inclusion of objective measures such as body mass index (BMI) and performance status, alongside metastatic distribution and tumor burden indicators, when evaluating determinants of early progression.

Accordingly, the present study examined clinical characteristics, baseline metastatic patterns, molecular features, and treatment-related variables in EGFR-mutant NSCLC patients receiving targeted therapy, with a focus on factors associated with early progression. This approach aligns with contemporary calls for personalized, data-driven care frameworks in EGFR-mutant NSCLC that integrate clinical phenotype, treatment context, and molecular profiling to optimize sequencing and surveillance strategies.

## 2. Methods

### 2.1. Study design

This study was approved by the Ethics Committee of Affiliated Hospital of Hebei University of Engineering. This single-center retrospective cohort study enrolled patients with EGFR-mutant NSCLC who received EGFR-targeted therapy at our institution between June 2022 and June 2025. Eligible patients were identified consecutively from the institutional electronic medical record system. All patients treated during the study interval who satisfied the inclusion criteria and did not meet any exclusion criterion were included in the analysis; no sampling procedure or matching strategy was used. The early-progression and nonearly-progression groups were generated by stratifying this cohort according to response evaluation criteria in solid tumors (RECIST)-defined progression status within 6 months after EGFR-TKI initiation, rather than by prespecified case-control matching. Eligible participants were required to have histologically or cytologically confirmed NSCLC, harbor a sensitizing EGFR alteration identified by a validated molecular assay performed on tumor tissue or plasma, have received an EGFR TKI as the primary targeted regimen in the relevant treatment line, have at least one measurable lesion according to RECIST version 1.1 and at least one posttreatment radiologic assessment enabling response categorization, and have complete baseline clinicopathologic and key laboratory/imaging data required for covariate analyses. Patients were excluded if they lacked confirmatory EGFR genotyping or had indeterminate/discordant results without adjudication, received targeted therapy for <4 weeks in the absence of documented radiographic progression or intolerance, initiated systemic anticancer therapy at an outside institution with insufficient documentation of regimen exposure and response assessments, had concomitant malignancies requiring active treatment during the study period, had primary endpoints not evaluable due to missing imaging or nonstandardized follow-up, or were lost to follow-up before the first response evaluation. The study was conducted in accordance with the Declaration of Helsinki and was approved by the institutional medical ethics committee; written informed consent was obtained from all participants.

### 2.2. Grouping criteria based on early disease progression

Patients were stratified according to early progression status. Early progression was defined a priori as the occurrence of progressive disease (PD) within 6 months after initiation of EGFR-targeted therapy, whereas the nonearly progression group comprised patients who did not develop PD within the first 6 months after treatment initiation. The 6-month cutoff was selected before data analysis because progression during the first 6 months of EGFR-TKI therapy represents a clinically meaningful pattern of early treatment failure that may reflect intrinsic resistance, aggressive baseline disease biology, or rapidly emerging resistant subclones. This time point also provides a practical landmark in retrospective real-world imaging data, as most patients undergo routine response assessment during the first several months of therapy. PD was adjudicated using the RECIST, version 1.1, and was defined by any of the following: a ≥20% increase in the sum of diameters of target lesions relative to the smallest sum of diameters recorded during follow-up (nadir), with an absolute increase of at least 5 mm; the appearance of one or more new lesions; and/or unequivocal progression of nontarget lesions on radiographic assessment. The date of PD was assigned as the earliest imaging time point at which RECIST v1.1 criteria for PD were met; for patients without documented PD, progression status was determined based on the most recent follow-up evaluation.

### 2.3. Data collection

Clinical and molecular data were retrospectively extracted from the institutional electronic medical record system and integrated with imaging and pathology databases using a standardized case report form. Variables were collected at baseline (defined as the date of index EGFR-TKI initiation) and, when applicable, during follow-up until documented disease progression, death, last clinical contact, or the end of the study period. Demographic and general clinical characteristics included age, sex, smoking status (never/former/current), BMI, Eastern Cooperative Oncology Group performance status (ECOG PS), and prespecified comorbidities (chronic obstructive pulmonary disease [COPD], cardiovascular disease, and diabetes mellitus). Tumor-related variables comprised histologic subtype, American Joint Committee on Cancer (AJCC) stage, burden of disease at treatment initiation (number of metastatic sites and number of target lesions), and baseline metastatic distribution (brain, liver, bone metastases, and pleural effusion). Molecular profiling variables included EGFR mutation status and predefined concomitant and resistance-related alterations, including TP53 co-mutation, baseline EGFR T790M, and mesenchymal–epithelial transition factor (MET) amplification, as documented in pathology reports and molecular testing records. Treatment-related variables captured the generation of the index EGFR TKI, line of targeted therapy, prior anticancer treatments (chemotherapy, radiotherapy, and immunotherapy), and treatment modification indicators (dose reduction and discontinuation because of adverse events [AEs]). Molecular testing results were abstracted from institutional pathology and molecular diagnostic reports generated as part of routine clinical care. Tissue-based testing was prioritized when available, while plasma-based testing was accepted when tissue was insufficient or unavailable; EGFR alterations, TP53 co-mutation, baseline EGFR T790M, and MET amplification were coded according to the original validated assay report and assay-specific reporting thresholds.

All radiographic assessments were reviewed according to routine institutional practice, and progression status was determined based on contemporaneous imaging reports and oncologist documentation, using the predefined criterion of PD occurring within 6 months for the early progression group. To ensure data quality, 2 investigators independently abstracted key variables, and discrepancies were resolved through rereview of source documents and consensus adjudication. De-identified data were stored in a password-protected dataset accessible only to study personnel.

### 2.4. Statistical analysis

All statistical analyses were performed using IBM SPSS Statistics, version 28.0 (IBM Corp.). Continuous variables were assessed for distributional characteristics and are presented as mean ± standard deviation for approximately normally distributed data or as median (interquartile range) for non-normally distributed data. Categorical variables are reported as counts and percentages. Because no matching strategy was used, baseline characteristics were compared between the early progression and nonearly progression groups as independent groups using the independent-samples *t* test for normally distributed continuous variables, the Mann–Whitney *U* test for non-normally distributed continuous variables, and the Pearson χ^2^ test for categorical variables; Fisher exact test was applied when expected cell counts were small. To identify factors associated with early progression, univariable logistic regression analyses were conducted with early progression (PD ≤ 6 months: yes/no) as the dependent variable. Candidate variables for multivariable logistic regression were selected based on a priori clinical relevance and univariable screening results, with attention to potential confounding by demographic factors, disease burden, metastatic distribution, molecular features, and treatment context. Age, sex, and ECOG PS were retained as clinically important baseline covariates, and highly overlapping variables were reviewed to reduce model instability. Results are reported as odds ratios (ORs) or adjusted odds ratios (aORs) with 95% confidence intervals (CIs) and two-sided *P* values. All tests were two-sided, and *P* < .05 was considered statistically significant.

## 3. Results

### 3.1. Baseline characteristics

A total of 156 patients with EGFR-mutant NSCLC were included and stratified into an early progression group (n = 52; PD) within 6 months of treatment initiation and a nonearly progression group (n = 104; no PD beyond 6 months). Between-group comparisons showed no statistically significant differences in age, sex distribution, ECOG PS, histologic subtype, AJCC stage, number of metastatic sites, or brain metastasis (all *P* > .05). In contrast, the early progression group had a significantly different smoking status distribution (χ^2^ = 10.69, *P* = .005), a lower BMI (*t* = −2.62, *P* = .010), and a higher prevalence of COPD (χ^2^ = 5.19, *P* = .023). Tumor burden indicators differed between groups, with a higher number of target lesions in the early progression group (median [interquartile rang] 4 [3–6] vs 3 [2–5]; *U* = 3414, *P* = .007). In addition, liver metastasis (χ^2^ = 7.96, *P* = .005), bone metastasis (χ^2^ = 12.86, *P* < .001), and pleural effusion (χ^2^ = 6.65, *P* = .010) were more frequent in the early progression group (Table 1).

**Table 1:** Baseline demographic and clinicopathologic characteristics.

Variable	Early progression (n = 52)	Nonearly progression (n = 104)	Test statistic	*P* value
Age (yr)	62.97 ± 8.32	60.36 ± 9.07	*t* = 1.79	.076
Male sex, n (%)	28 (53.8)	53 (51.0)	χ^2^ = 0.12	.734
Smoking status, n (%)				
Never	19 (36.5)	63 (60.6)	χ^2^ = 10.69	.005
Former	15 (28.8)	26 (25.0)		
Current	18 (34.6)	15 (14.4)		
BMI, kg/m^2^	23.36 ± 2.85	24.68 ± 3.25	*t* = −2.62	.010
ECOG PS, n (%)				
0	13 (25.0)	38 (36.5)	χ^2^ = 2.33	.311
1	33 (63.5)	58 (55.8)		
2	6 (11.5)	8 (7.7)		
COPD, n (%)	14 (26.9)	14 (13.5)	χ^2^ = 5.19	.023
Cardiovascular disease, n (%)	15 (28.8)	24 (23.1)	χ^2^ = 1.46	.227
Diabetes mellitus, n (%)	11 (21.2)	23 (22.1)	χ^2^ = 0.16	.687
Histology, n (%)				
Adenocarcinoma	42 (80.8)	93 (89.4)	χ^2^ = 1.76	.414
Squamous	7 (13.5)	8 (7.7)		
Other	3 (5.8)	3 (2.9)		
AJCC stage, n (%)				
III	7 (13.5)	14 (13.5)	χ^2^ = 0.00	1.000
IV	45 (86.5)	90 (86.5)		
Number of metastatic sites, median (IQR)	3 (2–4)	3 (2–4)	*U* = 3012 (*Z* = 1.16)	.237
Number of target lesions, median (IQR)	4 (3–6)	3 (2–5)	*U* = 3414 (*Z* = 2.67)	.007
Brain metastasis, n (%)	17 (32.7)	30 (28.8)	χ^2^ = 0.85	.356
Liver metastasis, n (%)	19 (36.5)	19 (18.3)	χ^2^ = 7.96	.005
Bone metastasis, n (%)	31 (59.6)	33 (31.7)	χ^2^ = 12.86	<.001
Pleural effusion, n (%)	18 (34.6)	17 (16.3)	χ^2^ = 6.65	.010

AJCC = American Joint Committee on Cancer, BMI = body mass index, COPD = chronic obstructive pulmonary disease, ECOG PS = Eastern Cooperative Oncology Group performance status, IQR = interquartile range.

### 3.2. Molecular and treatment profiles

Among molecular alterations, TP53 co-mutation was more frequent in the early progression group than in the nonearly progression group (57.7% vs 39.4%; χ^2^ = 4.76, *P* = .029). Baseline EGFR T790M was also observed more often in the early progression group (15.4% vs 2.9%), and the between-group difference was significant by Fisher exact test (OR = 9.27, *P* = .003). In contrast, MET amplification was uncommon and did not differ significantly between groups (7.7% vs 2.9%; Fisher exact OR = 3.58, *P* = .118). Regarding treatment characteristics, the distribution of EGFR-TKI generation differed significantly between groups (χ^2^ = 7.14, *P* = .028), with the early progression group showing a higher proportion of first-generation EGFR-TKI use (48.1% vs 30.8%) and a lower proportion of third-generation EGFR-TKI use (34.6% vs 55.8). In addition, the line of targeted therapy differed substantially, with early progression patients more frequently receiving EGFR-TKI as ≥second-line treatment (57.7% vs 25.0%; χ^2^ = 15.78, *P* < .001). By comparison, prior treatment exposures and treatment modification indicators were broadly comparable across groups. No significant differences were observed for prior chemotherapy (46.2% vs 39.4%; χ^2^ = 1.39, *P* = .238), prior radiotherapy (38.5% vs 35.6%; χ^2^ = 0.51, *P* = .475), or prior immunotherapy (17.3% vs 10.6%; χ^2^ = 2.54, *P* = .111). Similarly, rates of dose reduction (26.9% vs 20.2%; χ^2^ = 1.74, *P* = .187) and treatment discontinuation because of AEs (13.5% vs 9.6%; χ^2^ = 0.53, *P* = .467) did not differ significantly between the early progression and nonearly progression groups (Table 2).

**Table 2:** Molecular characteristics and treatment-related variables by progression group.

Variable	Early progression (n = 52)	Nonearly progression (n = 104)	Test statistic	*P* value
TP53 co-mutation, n (%)	30 (57.7)	41 (39.4)	χ^2^ = 4.76	.029
Baseline EGFR T790M, n (%)	8 (15.4)	3 (2.9)	Fisher exact (OR = 9.27)	.003
MET amplification, n (%)	4 (7.7)	3 (2.9)	Fisher exact (OR = 3.58)	.118
EGFR-TKI generation, n (%)				
First	25 (48.1)	32 (30.8)	χ^2^ = 7.14	.028
Second	9 (17.3	14 (13.5)		
Third	18 (34.6)	58 (55.8)		
Line of targeted therapy, n (%)				
First-line	22 (42.3)	78 (75.0)	χ^2^ = 15.78	<.001
≥Second-line	30 (57.7)	26 (25.0)		
Prior chemotherapy, n (%)	24 (46.2)	41 (39.4)	χ^2^ = 1.39	.238
Prior radiotherapy, n (%)	20 (38.5)	37 (35.6)	χ^2^ = 0.51	.475
Prior immunotherapy, n (%)	9 (17.3)	11 (10.6)	χ^2^ = 2.54	.111
Dose reduction, n (%)	14 (26.9)	21 (20.2)	χ^2^ = 1.74	.187
Treatment discontinuation due to AEs, n (%)	7 (13.5)	10 (9.6)	χ^2^ = 0.53	.467

AEs = adverse events, EGFR = epidermal growth factor receptor, EGFR-TKI = epidermal growth factor receptor tyrosine kinase inhibitor, MET = mesenchymal–epithelial transition factor, OR = odds ratio.

### 3.3. Univariable logistic regression for potential factors associated with early disease progression

In univariable logistic regression, current smoking versus never smoking was associated with higher odds of early progression (OR = 3.98, 95% CI = 1.69–9.36, *P* = .002), whereas higher BMI was inversely associated with early progression (per 1 kg/m^2^ increase: OR = 0.87, 95% CI = 0.78–0.97, *P* = .015). COPD was associated with early progression (OR = 2.82, 95% CI = 1.13–7.06, *P* = .027). Indicators of tumor burden and dissemination were also associated with early progression, including a higher number of target lesions (per 1 lesion: OR = 1.20, 95% CI = 1.02–1.40, *P* = .024), liver metastasis (OR = 2.58, 95% CI = 1.22–5.45, *P* = .013), bone metastasis (OR = 3.17, 95% CI = 1.59–6.30, *P* = .001), and pleural effusion (OR = 2.72, 95% CI = 1.28–5.80, *P* = .009). From the molecular and treatment domains, TP53 co-mutation (OR = 2.10, 95% CI = 1.05–4.19, *P* = .036) and baseline EGFR T790M (OR = 6.13, 95% CI = 1.55–24.20, *P* = .010) were associated with early progression. Compared with third-generation EGFR-TKIs, first-generation EGFR-TKI use was associated with increased odds of early progression (OR = 2.52, 95% CI = 1.24–5.13, *P* = .011). Receiving EGFR-TKI as ≥second-line therapy versus first-line showed the strongest association with early progression (OR = 4.09, 95% CI = 2.04–8.20, *P* < .001). Other baseline factors (sex, ECOG PS, cardiovascular disease, diabetes, histology, AJCC stage, number of metastatic sites, and brain metastasis), as well as prior treatments and treatment modification indicators (prior chemotherapy/radiotherapy/immunotherapy, dose reduction, and AE-related discontinuation), were not significantly associated with early progression in univariable analyses (all *P* > .05; Table 3).

**Table 3:** Univariable logistic regression for potential factors associated with early disease progression.

Predictor	Term	OR	95% CI	*P* value
Age (per 1-yr increase)	Age (per 1-yr increase)	1.03	1.00–1.07	.085
Male sex	Male (yes vs no)	1.12	0.58–2.19	.734
Smoking status	Smoking: current	3.98	1.69–9.36	.002
Smoking status	Smoking: former	1.91	0.85–4.33	.120
BMI (per 1 kg/m^2^ increase)	BMI (per 1 kg/m^2^ increase)	0.87	0.78–0.97	.015
ECOG PS	ECOG PS 1	1.66	0.78–3.56	.190
ECOG PS	ECOG PS 2	2.19	0.64–7.51	.212
COPD	COPD (yes vs no)	2.82	1.13–7.06	.027
Cardiovascular disease	Cardiovascular disease (yes vs no)	0.60	0.26–1.38	.229
Diabetes mellitus	Diabetes mellitus (yes vs no)	1.17	0.54–2.56	.687
Histology	Histology: other	2.22	0.55–9.00	.262
Histology	Histology: squamous	1.94	0.69–5.42	.207
AJCC stage	AJCC stage: IV	1.00	0.37–2.70	1.000
Number of metastatic sites (per 1 site)	Number of metastatic sites (per 1 site)	1.18	0.89–1.58	.246
Number of target lesions (per 1 lesion)	Number of target lesions (per 1 lesion)	1.20	1.02–1.40	.024
Brain metastasis	Brain metastasis (yes vs no)	1.20	0.60–2.40	.607
Liver metastasis	Liver metastasis (yes vs no)	2.58	1.22–5.45	.013
Bone metastasis	Bone metastasis (yes vs no)	3.17	1.59–6.30	.001
Pleural effusion	Pleural effusion (yes vs no)	2.72	1.28–5.80	.009
TP53 co-mutation	TP53 co-mutation (yes vs no)	2.10	1.05–4.19	.036
Baseline EGFR T790M	Baseline EGFR T790M (yes vs no)	6.13	1.55–24.20	.010
MET amplification	MET amplification (yes vs no)	2.80	0.60–13.12	.190
EGFR-TKI generation	EGFR-TKI generation: first	2.52	1.24–5.13	.011
EGFR-TKI generation	EGFR-TKI generation: second	2.07	0.81–5.29	.127
Line of targeted therapy	Line of therapy: ≥second-line	4.09	2.04–8.20	<.001
Prior chemotherapy	Prior chemotherapy (yes vs no)	1.32	0.68–2.56	.413
Prior radiotherapy	Prior radiotherapy (yes vs no)	1.13	0.57–2.25	.727
Prior immunotherapy	Prior immunotherapy (yes vs no)	1.77	0.69–4.54	.234
Dose reduction	Dose reduction (yes vs no)	1.46	0.67–3.20	.342
Treatment discontinuation due to AEs	Discontinuation due to AEs (yes vs no)	1.46	0.53–4.03	.468

AEs = adverse events, AJCC = American Joint Committee on Cancer, BMI = body mass index, CI = confidence interval, COPD = chronic obstructive pulmonary disease, ECOG PS = Eastern Cooperative Oncology Group performance status, EGFR = epidermal growth factor receptor, EGFR-TKI = epidermal growth factor receptor tyrosine kinase inhibitor, MET = mesenchymal–epithelial transition factor, OR = odds ratio.

### 3.4. Multivariable analysis: independent risk factors for early progression

In the multivariable logistic regression model adjusting for baseline characteristics, disease burden, molecular features, and treatment-related factors, several variables remained independently associated with early progression. Current smoking (vs never smoking) was associated with higher odds of early progression (aOR = 6.03, 95% CI = 1.66–21.97, *P* = .006). Greater tumor burden, reflected by a higher number of target lesions, was independently associated with early progression (per 1-lesion increase: aOR = 1.60, 95% CI = 1.16–2.22, *P* = .004). Specific metastatic patterns were also independently associated with early progression, including liver metastasis (aOR = 6.70, 95% CI = 1.89–23.68, *P* = .003), bone metastasis (aOR = 7.20, 95% CI = 2.41–21.48, *P* < .001), and pleural effusion (aOR = 3.69, 95% CI = 1.23–11.11, *P* = .020). Treatment-related factors showed strong independent effects: receiving EGFR-TKI as ≥second-line therapy (vs first-line) was associated with markedly increased odds of early progression (aOR = 12.41, 95% CI = 3.52–43.76, *P* < .001). Compared with third-generation EGFR-TKIs, the use of a first-generation EGFR-TKI remained associated with early progression (aOR = 3.26, 95% CI = 1.05–10.10, *P* = .040). Among molecular variables, MET amplification was independently associated with early progression (aOR = 15.87, 95% CI = 1.95–128.84, *P* = .010), while baseline EGFR T790M showed a borderline association after adjustment (aOR = 6.52, 95% CI = 0.92–46.14, *P* = .061). Higher BMI remained inversely associated with early progression (per 1 kg/m^2^ increase: aOR = 0.83, 95% CI = 0.71–0.97, *P* = .018). Age, sex, and ECOG PS were not independently associated with early progression in the adjusted model (all *P* > .05; Table 4).

**Table 4:** Multivariable logistic regression for independent factors associated with early disease progression.

Predictor	aOR	95% CI	*P* value
Smoking: former	3.26	0.99–10.68	.051
Smoking: current	6.03	1.66–21.97	.006
ECOG PS: 1	0.98	0.32–2.99	.974
ECOG PS: 2	0.62	0.11–3.59	.589
EGFR-TKI generation: first	3.26	1.05–10.10	.040
EGFR-TKI generation: second	1.49	0.28–8.00	.640
Line of targeted therapy: ≥second-line	12.41	3.52–43.76	<.001
Age (per 1-yr increase)	1.04	0.98–1.11	.214
Male sex (yes vs no)	1.08	0.41–2.86	.880
BMI (per 1 kg/m^2^ increase)	0.83	0.71–0.97	.018
Number of target lesions (per 1 lesion)	1.60	1.16–2.22	.004
Liver metastasis (yes vs no)	6.70	1.89–23.68	.003
Bone metastasis (yes vs no)	7.20	2.41–21.48	<.001
Pleural effusion (yes vs no)	3.69	1.23–11.11	.020
Baseline EGFR T790M (yes vs no)	6.52	0.92–46.14	.061
MET amplification (yes vs no)	15.87	1.95–128.84	.010

aOR = adjusted odds ratio, BMI = body mass index, CI = confidence interval, ECOG PS = Eastern Cooperative Oncology Group performance status, EGFR = epidermal growth factor receptor, EGFR-TKI = epidermal growth factor receptor tyrosine kinase inhibitor, MET = mesenchymal–epithelial transition factor.

## 4. Discussion

In this retrospective cohort of patients with EGFR-mutant NSCLC receiving targeted therapy, early disease progression within 6 months was associated with a distinct pattern of clinical burden, metastatic distribution, and molecular/treatment-relatedfeatures. In adjusted analyses, current smoking, lower BMI, a higher number of target lesions, liver metastasis, bone metastasis, pleural effusion, receipt of EGFR-TKI as ≥second-line therapy, use of a first-generation EGFR-TKI (vs third-generation),and MET amplification remained independently associated with early progression, while baseline EGFR T790M showed a borderline association after adjustment. Collectively, these findings support the concept that early progression under EGFR-directed therapy is not driven by a single factor, but rather by the interaction between baseline disease aggressiveness, host condition, and actionable or bypass molecular alterations.

The higher prevalence of TP53 co-mutation in the early progression group and its association with early progression in univariable analysis are consistent with accumulating evidence that TP53 alterations define a clinically higher-risk subgroup within EGFR-mutant disease. Recent studies have reported inferior outcomes among patients harboring concurrent EGFR and TP53 mutations treated with EGFR-TKIs, supporting TP53 as a marker of unfavorable biology and shortened time-to-treatment failure.^[11,12]^ Mechanistically, TP53 dysfunction may facilitate genomic instability, lineage plasticity, and tolerance to oncogene inhibition, thereby enabling more rapid emergence of resistant clones. These biological considerations provide a plausible explanation for the observed enrichment of TP53 co-mutation among early progressors. In the multivariable model, TP53 was not retained as an independent factor once tumor burden, metastatic pattern, and treatment line/generation were accounted for, which may reflect partial mediation by disease severity at treatment initiation or limited power to detect an independent effect after adjustment. Baseline EGFR T790M was more frequent in early progressors and strongly associated with early progression in univariable analysis; however, the association attenuated to borderline significance after multivariable adjustment. This pattern suggests that baseline T790M may contribute to early resistance risk, but that its apparent effect is sensitive to confounding by treatment context and disease burden. Evidence from translational analyses of osimertinib-treated populations indicates that resistant subclones and heterogeneous resistance mechanisms can shape early failure, particularly when resistant alterations coexist with additional genomic events.^[13,14]^ Thus, baseline T790M may function as a proxy for clonal complexity or preexisting resistant subpopulations rather than an isolated driver of early progression in every patient.

MET amplification warrants particular attention because it emerged as an independent factor in the multivariable model despite low absolute frequency. MET amplification is a well-recognized bypass mechanism that can activate downstream signaling despite EGFR blockade and is repeatedly identified in resistance profiling studies.^[15,16]^ The finding that MET amplification was independently associated with early progression is clinically relevant because MET-driven resistance is potentially targetable. In this context, prospective evidence supports combined EGFR and MET inhibition in EGFR-mutated NSCLC with MET amplification; for example, tepotinib plus osimertinib demonstrated meaningful activity in patients with MET amplification as a resistance mechanism.^[17]^ Although the present study did not evaluate postprogression treatment efficacy, the observed association underscores the rationale for early molecular characterization (including MET assessment) when rapid progression is observed, and for considering MET-directed strategies where available and appropriate.

A consistent finding across univariable and multivariable analyses was the strong association between ≥second-line use of EGFR-TKI and early progression. This relationship is clinically plausible because later-line targeted therapy often occurs after prior systemic treatments and clonal evolution, potentially increasing the prevalence of resistant subclones and diminishing dependence on EGFR signaling. Furthermore, patients treated in later lines may have more advanced disease burden or compromised physiological reserve, which could contribute to early failure. While the multivariable model adjusted for multiple baseline burden indicators, residual confounding is likely in retrospective datasets, and treatment line may also capture unmeasured features such as prior response patterns, time-varying tumor heterogeneity, and differences in molecular testing strategies. The association between first-generation EGFR-TKI use (vs third-generation) and early progression is aligned with contemporary guideline-based practice that favors third-generation EGFR-TKIs (notably osimertinib) as preferred first-line therapy for classical activating EGFR mutations, particularly given central nervous system activity and improved efficacy endpoints reported across studies and reflected in practice guidelines.^[18]^ In this cohort, the early progression group had a lower proportion of third-generation EGFR-TKI use and a higher proportion of first-generation use, which may reflect treatment access, temporal prescribing patterns across the study period, or clinical selection. The persistence of an association after adjustment suggests that TKI generation may contribute independently to early failure risk in real-world practice, although confounding by indication cannot be fully excluded.

Indicators of tumor burden and dissemination were prominent determinants of early progression. A higher number of target lesions was independently associated with early progression, supporting the notion that measurable disease volume and heterogeneity reduce the probability of durable control under monotherapy. In addition, liver metastasis, bone metastasis, and pleural effusion remained independently associated with early progression. Real-world datasets have similarly identified metastatic patterns such as liver involvement as adverse prognostic features in EGFR-mutant NSCLC treated with targeted therapy.^[19]^ Biologically, liver metastasis may represent more aggressive tumor phenotypes and immunologically distinct niches, while pleural effusion may indicate diffuse pleural involvement and high tumor burden that can be difficult to control with systemic therapy alone. Bone metastasis is also associated with increased morbidity and may reflect advanced dissemination and complex tumor–microenvironment interactions.

Current smoking was independently associated with early progression. While EGFR mutations are more common in never-smokers, smoking exposure remains clinically meaningful among EGFR-mutant patients and may influence tumor biology, co-mutation profiles, and pharmacodynamic vulnerability. The strong association in the adjusted model suggests that smoking status may capture features of tumor aggressiveness or concurrent genomic complexity not fully represented by the measured molecular variables. This finding supports the inclusion of smoking history in risk stratification for early progression and highlights the importance of standardized documentation of tobacco exposure in real-world datasets. Lower BMI was independently associated with early progression. BMI may reflect nutritional status, systemic inflammation, or cancer-associated cachexia, all of which can affect tolerance to therapy and overall disease trajectory. Recent evidence indicates that weight loss during osimertinib therapy is common and is associated with worse survival outcomes in EGFR-mutant metastatic disease, suggesting that host metabolic status is clinically relevant in this setting.^[20]^ While BMI is a crude surrogate and cannot distinguish sarcopenia from adiposity, the observed inverse association supports the integration of nutritional and body composition assessment in future studies and suggests that early supportive interventions may be appropriate for high-risk patients.

Several limitations should be considered when interpreting these findings. First, the retrospective single-center cohort design introduces potential selection bias, incomplete capture of confounders, and heterogeneity in diagnostic work-up and treatment decisions over time. Although patients were consecutively screened and all eligible patients were included, no matching strategy was used; therefore, baseline imbalances and residual confounding by indication, treatment access, prior therapy, and unmeasured disease biology cannot be fully excluded. Second, the definition of early progression (PD ≤ 6 months) was selected a priori as a clinically meaningful landmark for early treatment failure, but it may still be influenced by assessment intervals and imaging frequency. Third, molecular characterization was limited to available variables; unmeasured co-alterations (e.g., additional bypass pathways) and differences in assay platforms and thresholds may have contributed to misclassification. Fourth, the low prevalence of certain alterations (notably MET amplification and baseline T790M) resulted in wide CIs, and effect estimates should be interpreted cautiously. Finally, external validity may be limited by the single-institution setting, underscoring the need for independent validation in larger, multicenter cohorts.

## 5. Conclusion

In this cohort of EGFR-mutant NSCLC patients receiving targeted therapy, early progression (PD ≤ 6 months) was independently associated with current smoking, lower BMI, higher target lesion count, liver and bone metastases, pleural effusion, ≥second-line EGFR-TKI use, first-generation EGFR-TKI exposure, and MET amplification. Baseline EGFR T790M showed a borderline association after adjustment. These findings support risk-adapted monitoring and biomarker-informed treatment optimization.

## Author contributions

**Conceptualization:** Xiaoyan Guo, Likai Liu, Hongyan Yu, Shifeng Zhao, Chunyan Wang, Xiansheng Wang.

**Data curation:** Xiaoyan Guo, Likai Liu, Hongyan Yu, Shifeng Zhao, Chunyan Wang, Xiansheng Wang.

**Formal analysis:** Xiaoyan Guo, Likai Liu, Hongyan Yu, Shifeng Zhao, Chunyan Wang, Xiansheng Wang.

**Funding acquisition:** Xiansheng Wang.

**Investigation:** Xiansheng Wang.

**Writing – original draft:** Xiansheng Wang.

**Writing – review & editing:** Xiansheng Wang.
